# Association of objectively measured lifting load with low-back pain, stress, and fatigue: A prospective cohort study

**DOI:** 10.5271/sjweh.4127

**Published:** 2024-01-01

**Authors:** Rúni Bláfoss, Per Aagaard, Thomas Clausen, Lars L Andersen

**Affiliations:** 1 National Research Centre for the Working Environment, Copenhagen, Denmark.; 2 Research Unit for Muscle Physiology and Clinical Biomechanics, Department of Sports Science and Clinical Biomechanics, University of Southern Denmark, Odense, Denmark.; 3 Department of Health Sciences and Technology, Aalborg University, Aalborg, Denmark.

**Keywords:** biopsychosocial, exposure–response, model, musculoskeletal disease, occupational group, occupational lifting, occupational stress, work, working condition

## Abstract

**Objectives:**

Limited knowledge exists about the association of lifting loads on a daily basis with physical and mental symptoms among warehouse workers. This study investigated associations between objectively measured lifting load and low-back pain (LBP), mental stress, and bodily fatigue after work and the following morning.

**Methods:**

Warehouse workers (N=85) from the retail industry replied to daily questionnaires before and after work for 21 days about LBP intensity, mental stress, and bodily fatigue (outcome, all scales 0–10). We assessed lifting exposure using company records from the warehouse logistic systems on total lifting load (kg) per workday. Associations between variables were tested using linear mixed models with repeated measures controlling for relevant confounders.

**Results:**

Mean daily lifting load was 1667.2 kg (range: 0–9998.4 kg). Compared to lifting 0–499 kg during a workday, lifting 500–1999 kg was associated with 0.59 points [95% confidence interval (CI) 0.10–1.08] elevated LBP intensity after work, while lifting ≥5000 showed a higher LBP intensity of 1.26 points (95% CI 0.48–2.03). LBP intensity remained elevated the following morning. Lifting ≥5000 kg was associated with higher mental stress after work of 0.74 points (95% CI 0.10–1.37), while no association was observed for bodily fatigue.

**Conclusions:**

Higher daily lifting loads were associated with higher LBP intensity after work and the following morning. These findings suggest that warehouses should consider the daily lifting loads when organizing warehouse work to prevent development of LBP, eg, using company records to provide a more equal distribution of daily lifting loads between workers.

Modern warehouses in the retail industry use digital logistics systems, which instruct the warehouse workers about what merchandise to pick through a headset or a vest mounted with a speaker. Manually picking and lifting merchandise from shelves/stands to pallets constitutes a large part of the daily activities warehouse workers perform (1). The warehouses hold company records containing information on the type and load (kg) of merchandises each worker handles, which enables the calculation of total lifting load during the working day (2). This information could be used to organize warehouse work to improve the physical working environment.

Occupational lifting entails a risk of developing low-back pain (LBP) (3, 4), which is among the most prevalent work-related musculoskeletal disorders (MSD) (5). In fact, MSD account for approximately half of absences from work and represent an overall estimated cost of work-related MSD of up to 2% reduced gross domestic product in the individual EU states (6). Furthermore, workers attending work despite feeling unwell or in pain also cost workplaces and societies due to reduced work productivity (presenteeism) (6). Additionally, a recent report from the Danish Health Authority shows that LBP itself produces a loss of productivity in Denmark of ~€2.7 billion due to absence from work, early disability and death compared to people without LBP (7). Using objective measures of load exposure, cumulative lifting loads, a work-related risk factor for developing LBP, has been found to increase both day-to-day and long-term development of LBP (8, 9). Besides increasing the risk of LBP/MSD, physically demanding work (including occupational lifting) is associated with higher levels of bodily fatigue (10, 11), which in turn represents a risk factor for developing pain (12). In turn, both musculoskeletal pain (including LBP) and bodily fatigue are significant predictors of future sickness absence (13, 14). Thus, examining the potential detrimental effects of occupational lifting is useful for increasing our current knowledge about how to organize manual work to prevent MSD.

Both physical and psychosocial job demands are important determinants of employee health and well-being (15). High physical and/or psychosocial job demands can increase the perception of strain (15), which is a known predictor of musculoskeletal pain (16). Perceived mental stress represents a psychological symptom for strain. Perceived stress can affect both physical and psychological well-being by increasing the risk of developing LBP (17) as well as depression and anxiety (18). Physically demanding occupations may, besides negatively affecting physical health, have detrimental effects on psychological factors as well, eg, leading to increased risk of mental stress (19). Consequently, high levels of work-related strain representing both physical and psychosocial job demands have been associated with higher odds of back pain (20). Thus, it is important to consider both physical and psychosocial job demands in order to prevent high strain.

Despite representing a large and highly global industry, little is known about the physical working environment among warehouse workers in the retail industry. In 2018, warehouse and transport workers in Denmark reported a high degree of occupational lifting and musculoskeletal pain (1). Out of 74 different job groups, warehouse and transport workers in Denmark ranked the 13^th^ highest in relation to physical work demands (21) and 37^th^ highest in relation to psychosocial work demands (22). However, limited evidence exists about associations between objectively measured occupational lifting loads in warehouses and health outcomes. The majority of studies investigating the association between physical job demands and health outcomes used questionnaires to quantify the physical exposures (3, 23, 24). However, assessing physical exposures and outcomes using questionnaire data entails a number of methodological limitations, which may bias the results, eg, by common-method variance (25). Using company records to objectively estimate physical exposures is a relatively cost-effective alternative that has previously shown promising results when examining scaffolding work (26). Furthermore, an exposure–response association between occupational lifting load quantified by company records and LBP intensity was observed among supermarket workers (9). Nonetheless, limited evidence exists about the association between occupational lifting and mental stress and fatigue.

The present study aimed to investigate the association between occupational daily lifting loads and LBP intensity, mental stress, and bodily fatigue after work and the following morning. Our primary hypothesis was that higher lifting loads would be associated with higher LBP intensity after work in an exposure–response manner. The secondary hypotheses were that higher daily lifting loads would be associated with elevated bodily fatigue in an exposure–response manner after work and LBP intensity and bodily fatigue would show the same pattern the following morning. Lastly, we hypothesized that higher lifting loads would be associated with elevated mental stress levels after work and the following morning, although not necessarily in a strong linear exposure–response fashion, given that psychosocial stressors are likely influenced by many factors besides lifting load.

## Methods

### Study design

This study is part of a 1-year prospective cohort study investigating the association between occupational lifting loads and work-related symptoms among retail industry warehouse workers in Denmark (2). Compared to our study protocol (2), several amendments have been conducted due to recruitment challenges and the COVID-19 pandemic from 2020–2022. These amendments are described in the supplementary material, www.sjweh.fi/article/4127.

**Figure 1:**
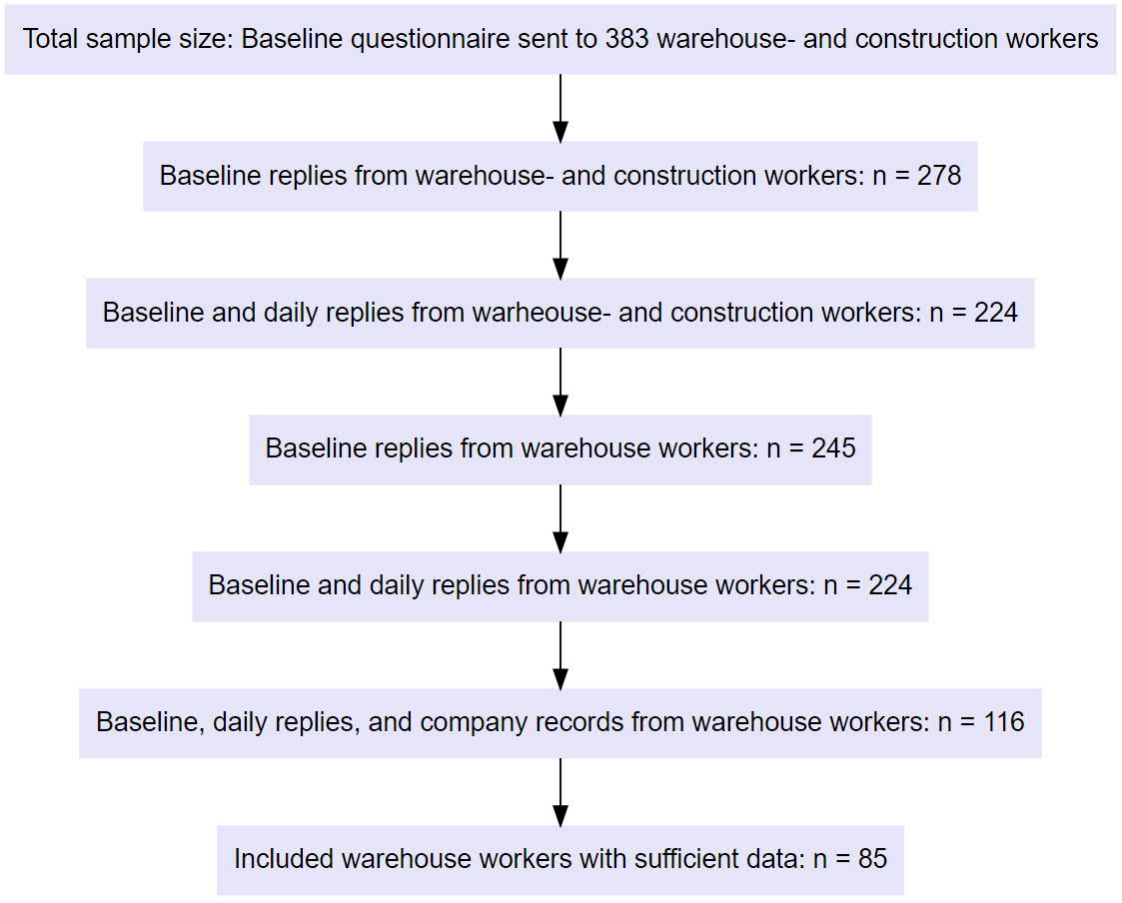
Study flow chart comprising the total subject sample.

The present investigation is a 3-week prospective cohort study with repeated measures combining company records of daily lifted merchandise per warehouse worker (exposure) with day-to-day information (twice-daily before and after work) about LBP intensity, mental stress, and bodily fatigue (outcomes, scales from 0–10). Daily questionnaires were sent for 21 days by SMS text messages in 12-hour intervals in the form of before and after work questionnaires, eg, at 06:00 and 18:00 hours each day. The time schedule of the daily questionnaires was chosen based on the workers’ working schedules. Preceding the 3-week observation period, the warehouse workers replied to a baseline questionnaire about physical and psychosocial working environment, general information, lifestyle, and health factors. Data collection spanned from September 2021 to March 2022. Reporting of the study follows the STROBE guidelines for observational studies (27). Figure 1 illustrates the flowchart of the study.

### Participants

We invited 383 warehouse and construction workers to participate in the study, of which 278 replied to the baseline questionnaire (response percentage: 72.6%) (figure 1). Participants were included if (i) working ~30 hours/week or more in a retail industry warehouse, (ii) ≥18 years old, (iii) able to read and understand Danish or English, and (iv) replying to the baseline and 3-week daily questionnaires. In the present study, we only included participants included in the company records with information about total lifting load (kg) during the workday over the course of the 3-week observation period (N=85). The included warehouse workers were employed in ten different warehouse terminals affiliated in five different retail chains in Denmark. Some warehouse sites packed merchandise for supermarkets/hypermarkets, while other sites handled products of personal hygiene, clothes, household equipment, and bread. Leaders at the warehouses provided phone numbers on their employees who worked with stocking of merchandise and were willing to participate. We then invited the workers to participate in the web-based baseline questionnaire sent via a SMS text message containing a web-link to the questionnaire. Beforehand, the leaders informed us whether the workers should receive the questionnaires in Danish or English. The 3-week period started two weeks subsequent to receiving the baseline questionnaire. Table 1 provides information about the study sample.

**Table 1:** Lifting load is based on company records. The table is divided into information collected at baseline and during the 3-week period. [N=number of participants (baseline questionnaire) and total observations of daily lifting load (3-week period) included in the analyses; SD=standard deviation; BMI=body mass index].

Participant characteristics		N	%	Mean	SD
**Baseline**					
Age		85		38.2	12.7
Sex		83			
	Men	54	65.1		
	Women	29	34.9		
BMI (kg/m^2^)		83		25.0	4.4
Smoking		83			
	Yes	26	31.3		
	No	57	68.7		
Physical activity during leisure		83			
	Reading, watching TV, or other sedentary activities	24	28.9		
	Walking, biking, or other light activities ≥4 hours/week	33	39.8		
	Sports, heavy gardening, or similar ≥4 hours/week	17	20.5		
	Vigorous exercise and sports several times per week	9	10.8		
Work ability (0–10)		83		7.9	1.6
Physically demanding work (0–10)		83		7.9	1.9
Employment (years)		83		9.9	11.3
Low-back pain intensity at baseline (0–10)		83		4.8	2.7
Chronic low-back pain		83			
	Yes	36	43.4		
	No	47	56.6		
Stress within the past 2 weeks at baseline		83			
	All the time	7	4.8		
	Often	16	21.7		
	Sometimes	20	36.1		
	Rarely	28	16.9		
	Never	12	20.5		
**3-week period**					
Manual lifting load per workday (kg)		85		1667.2	1886.7
Total observations of manual lifting load (kg) for weight categories		941			
	0–499	439	46.7		
	500–1999	143	15.2		
	2000–3499	118	12.5		
	3500–4999	191	20.3		
	≥5000	50	5.3		
Working hours per workday		85		7.7	0.9

### Ethical aspects

Danish legislation does not require ethical approval to be attained for scientific questionnaire-based studies nor is informed consent from study participants needed. Nonetheless, all questionnaire data were stored on a secure server and handled anonymously. A data manager de-identified the data before the researchers initiated the data analyses. The project is registered at the Danish Data Protection Agency.

Prior to receiving the baseline questionnaire, workers received written and oral information about the project. When receiving the baseline questionnaire, the SMS text message comprised a brief explanation about the baseline questionnaire and a web-link directing to the questionnaire survey. When entering the web-link, the first page of the baseline questionnaire included a thorough description of the project, their rights as participant, and contact information on the project leader.

### Company records (exposure)

During the 3-week study period, the leaders at the warehouse sites provided company records with information on the merchandise handled by each warehouse workers. The level of detail varied between retail chains where some warehouses delivered total daily lifting loads per warehouse workers (kg), while other provided type, weight, and quantity of all merchandise handled per worker. In these detailed company records, the total daily lifting load represented the sum of the load of all merchandises manually handled.

Before initiating the statistical analyses, the principal investigator examined all the detailed company records to remove loads not manually lifted. This was conducted by agreeing on some criteria with the site leaders on unrealistically high values of individual lifting loads and quantity of merchandise.

### Daily questionnaires (outcome)

SMS text messages were sent to all participants via a web-based survey platform (SurveyXact) containing a short text and a web-link directing the user to the survey questions. Participants replied to questions about LBP intensity, mental stress, and bodily fatigue. We measured LBP intensity using the validated numeric rating scale (NRS) of 0–10 by asking “How much pain do you experience in your low back this morning?”, where 0 represented “no pain at all” while 10 was “worst imaginable pain” (28). Symptoms for daily variation in mental stress level was examined by the question “How stressed do you feel this morning?”, answering on a 0–10 scale with 0 being “not stressed at all” and 10 being “maximally stressed”. We measured fatigue using the validated NRS–Fatigue of 0–10 with the question “How tired are you in the body this morning?”, where the participants replied by choosing a number between 0–10, where 0 was “not tired at all” and 10 was “completely exhausted” (29, 30). In the morning, all questions ended with “… this morning”, while after the working day questions ended with “… this evening”. For participants on night shifts working from the evening until during the night, the question before work ended with “… this afternoon”, while we framed the question after work ‘… this morning”. Throughout the article, we refer the “before work” estimates/replies as “the following morning”, also among workers on nightshifts.

### Potential confounders

We adjusted the analyses for relevant confounders. In the minimally adjusted models, we adjusted for sex (categorical: man, woman), age (continuous), employment with lifting work (continuous), daily working hours, job title (eg, picker, packer, supervisor) (categorical), LBP intensity during the past four weeks (continuous, NRS 0–10), chronic LBP defined as LBP several times weekly during the past 3 months (categorical: yes, no), and perceived stress within the last two weeks prior to baseline (categorical: all the time, often, sometimes, rarely, never). In the fully adjusted models, we additionally adjusted for the following lifestyle- and work-related factors: smoking (categorical: yes, no), leisure-time physical activity (categorical: sedentary, light, moderate, vigorous), body mass index (BMI, kg/m^2^, continuous), influence at work (categorical), access to necessary work tools (categorical), role clarity (categorical), guidance (categorical), community and cohesion between colleagues (categorical), recognition (categorical), respectful relationship between leader and employees (categorical), and fairness (categorical). The reply options for influence at work, access to necessary work tools, role clarity, guidance, community and cohesion, recognition, respectful relationship, and fairness were “to a very large extent”, to a large extent”, “somewhat”, “to a small extent”, “to a very small extent”.

### Statistical analyses

Linear mixed models with repeated measures (Proc Mixed, SAS version 9.4, SAS Institute, Cary, NC, USA) tested the association between variables. Working teams were entered as a random factor to account for clustering. Participant was entered as a repeated factor (21 days) with an autoregressive covariance structure. Outcome variables were LBP, mental stress, and bodily fatigue after work as well as the following morning/day before work (depending on the work schedule). The analyses were adjusted for the confounders mentioned previously. The explanatory factor was total load (kg) (categorical variable). Results are reported as least square means (LSM) and differences in LSM and 95% confidence intervals (CI). An alpha level of P<0.05 was considered as statistically significant.

## Results

### Participant characteristics

Mean age of participants was 38.2 (SD 12.7) years and 65.1% were men. On average, they tended to be overweight with a mean BMI of 25.0 (SD 4.4) kg/m^2^, and 28.9% were sedentary during leisure. At baseline, participants reported a mean LBP intensity of 4.8 (SD 2.7) points (NRS) during the preceding 4 weeks, and 43.4% had experienced LBP several times weekly during the past three months (ie, chronic LBP). On average, participants worked 7.7 (SD 0.9) hours per workday and manually lifted 1667.2 (SD 1886.7) kg per workday (range: 0–9998.4 kg).

### Low-back pain

**Figure 2:**
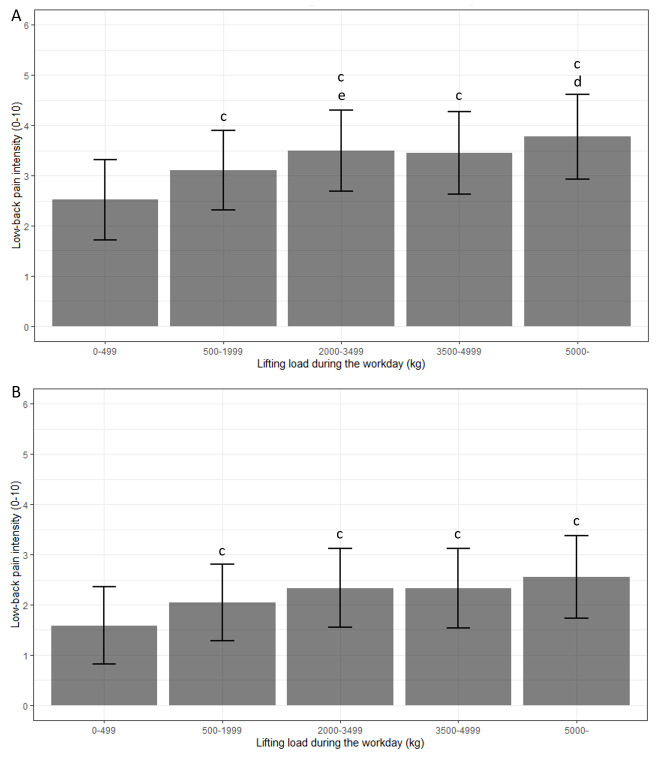
Association between total lifting load during the workday and low-back pain intensity (absolute estimates) after work (A) and the following morning (B) in the fully adjusted model (NRS 0–10). ^c^ Statistically significant different from 0–499 kg (reference). ^d^ Statistically significant different from 500–1999 kg. ^e^ Tendency towards a statistically significant difference from 500–1999 kg (P=0.062).

Higher total lifting loads were associated with higher LBP intensity (table 2, figure 2), although not in a linear exposure–response fashion. Compared to lifting 0–499 kg, the minimally adjusted model showed that lifting 500–1999 kg was associated with an elevated LBP intensity of 0.52 points (95% CI 0.03–1.00) after work, while lifting ≥5000 kg was associated with an elevated LBP intensity of 1.20 points (95% CI 0.43–1.96). The following morning, LBP was elevated in the minimally adjusted model by 0.43 points (95% CI 0.02 –0.84) when lifting 500–1999 kg the preceding day, while lifting ≥5000 kg was associated with an elevated LBP intensity the following morning by 0.91 points (95% CI 0.21–1.60).

**Table 2:** Associations between daily lifting load and low-back pain intensity after the workday and in the following morning. [LSM=least squares means; CI=confidence intervals].

Lifting load (kg)		N	Minimally adjusted ^a^			P-value	Fully adjusted ^b^			P-value
			LSM		LSM differences		LSM		LSM differences	
			Estimates		Difference (95% CI)		Estimates		Difference (95% CI)	
After work										
	0–499	439	2.03		Reference		2.52		Reference	
	500–1999	143	2.55		0.52 (0.04–1.00) ^c^	0.034	3.10		0.59 (0.10–1.08) ^c^	0.019
	2000–3499	118	2.95		0.92 (0.34–1.49) ^c, e^	0.002	3.50		0.98 (0.40–1.57) ^c, e^	0.001
	3500–4999	191	2.91		0.88 (0.24–1.51) ^c^	0.007	3.45		0.93 (0.28–1.58) ^c^	0.005
	≥5000	50	3.22		1.19 (0.43–1.96) ^c, d^	0.002	3.77		1.26 (0.48–2.03) ^c, d^	0.002
The following morning										
	0–499	439	1.20		Reference		1.59		Reference	
	500–1999	143	1.64		0.43 (0.02–0.84) ^c^	0.038	2.04		0.46 (0.04–0.87) ^c^	0.031
	2000–3499	118	1.93		0.73 (0.23–1.22) ^c^	0.005	2.34		0.75 (0.24–1.25) ^c^	0.004
	3500–4999	191	1.92		0.72 (0.17–1.27) ^c^	0.010	2.32		0.73 (0.18–1.29) ^c^	0.010
	≥5000	50	2.11		0.91 (0.21–1.60) ^c^	0.011	2.56		0.97 (0.27–1.67) ^c^	0.007

^a^ Minimally adjusted model: Adjusted for age, sex, job title, years of employment, daily working hours, low-back pain intensity the preceding 4 weeks prior to baseline, chronic low-back pain, and perceived stress during the two weeks prior to baseline. ^b^ Fully adjusted model: Minimally adjusted model + BMI, leisure-time physical activity, smoking status, influence at work, access to work tools, role clarity, guidance, community and cohesion between colleagues, recognition, respectful relationship between leader and employees, and fairness. ^c^ Statistically significant different from 0–499 kg (reference). ^d^ Statistically significant different from 500–1999 kg. ^e^ Tendency towards a statistically significant difference from 500–1999 kg (P=0.06).

Compared to lifting 0–499 kg, the fully adjusted model showed that lifting 500–1999 kg was associated with a higher LBP intensity of 0.59 points (95% CI 0.10–1.08) after work, while lifting ≥5000 kg showed an elevated LBP intensity of 1.26 points (95% CI 0.48 –2.03) (table 2). The following morning, LBP intensity was 0.46 points higher (95% CI 0.04–0.87) when lifting 500–1999 kg the day before, while lifting ≥5000 kg was associated with an elevated LBP intensity of 0.97 points the following morning (95% CI 0.27–1.67).

### Mental stress

Lifting ≥5000 kg during the workday was associated with higher mental stress after work by 0.74 points (95% CI 0.10–1.37) in the minimally adjusted model and 0.72 points (95% CI 0.08–1.36) in the fully adjusted model compared to lifting 0–499 kg (table 3).

**Table 3:** Associations between daily lifting load and mental stress after the workday and on the following morning. [LSM=least squares means; CI=confidence intervals].

Lifting load (kg)		N	Minimally adjusted ^a^			P-value	Fully adjusted ^b^			P-value
			LSM		LSM differences		LSM		LSM differences	
			Estimates		Difference (95% CI)		Estimates		Difference (95% CI)	
After work										
	0–499	439	1.17		Reference		1.24		Reference	
	500–1999	143	1.28		0.11 (-0.29–0.50)	0.596	1.36		0.12 (-0.27–0.52)	0.540
	2000–3499	118	1.35		0.18 (-0.30–0.66)	0.459	1.43		0.19 (-0.29–0.67)	0.439
	3500–4999	191	1.21		0.04 (-0.49–0.58)	0.875	1.27		0.03 (-0.50–0.57)	0.900
	≥5000	50	1.91		0.74 (0.10–1.37)^c–f^	0.024	1.96		0.72 (0.08–1.36)^c–f^	0.028
The following morning										
	0–499	439	0.44		Reference		0.65		Reference	
	500–1999	143	0.65		0.21 (-0.18–0.59)	0.289	0.88		0.23 (-0.16–0.62)	0.246
	2000–3499	118	1.05		0.60 (0.12–1.08)^c,d,f,g^	0.014	1.28		0.63 (0.15–1.11) ^c,d,f,g^	0.010
	3500–4999	191	0.60		0.16 (-0.37–0.68)	0.562	0.83		0.18 (-0.35–0.71)	0.511
	≥5000	50	0.46		0.02 (-0.64–0.68)	0.955	0.69		0.04 (-0.63–0.70)	0.911

^a^ Minimally adjusted model: Adjusted for age, sex, job title, years of employment, daily working hours, low-back pain intensity the preceding 4 weeks prior to baseline, chronic low-back pain, and perceived stress during the two weeks prior to baseline. ^b^ Fully adjusted model: Minimally adjusted + BMI, leisure-time physical activity, smoking status, influence at work, access to work tools, role clarity, guidance, community and cohesion between colleagues, recognition, respectful relationship between leader and employees, and fairness. ^c^ Statistically significant different from 0–499 kg (reference). ^d^ Statistically significant different from 500–1999 kg. ^e^ Statistically significant different from 2000–3499 kg. ^f^ Statistically significant different from 3500–4999 kg. ^g^ Statistically significant different from ≥5000 kg.

The following morning, the minimally adjusted model showed a statistically significant association between lifting 2000–3499 kg and higher mental stress by 0.60 points (95% CI 0.12–1.08), with the fully adjusted model also showing mental stress to be 0.63 points higher (95% CI 0.15–1.11) (table 3).

### Bodily fatigue

No association was observed between lifting loads and bodily fatigue. However, a trend to reach statistical significance was observed in the minimally adjusted model (0.74 points (95% CI -0.08–1.55), P=0.076) and in the fully adjusted model (0.75 points (95% CI -0.07– 1.57), P=0.071) between lifting ≥5000 kg during work and elevated levels of bodily fatigue in the following morning (table 4).

**Table 4:** Association between daily lifting load and bodily fatigue after work and on the following morning. [LSM=least squares means; CI=confidence intervals].

Lifting load (kg		N	Minimally adjusted ^a^			P-value	Fully adjusted ^b^			P-value
			LSM		LSM differences		LSM		LSM differences	
			Estimates		Difference (95% CI)		Estimates		Difference (95% CI)	
After work										
	0–499	439	3.31		Reference		2.90		Reference	
	500–1999	143	3.54		0.24 (-0.26–0.74)	0.355	3.16		0.26 (-0.24–0.76)	0.307
	2000–3499	118	3.61		0.30 (-0.30–0.90)	0.327	3.20		0.30 (-0.29–0.90)	0.317
	3500–4999	191	3.67		0.36 (-0.30–1.02)	0.285	3.25		0.35 (-0.31–1.01)	0.302
	≥5000	50	3.94		0.63 (-0.16–1.43)	0.117	3.53		0.63 (-0.16–1.42)	0.118
The following morning										
	0–499	439	2.22		Reference		2.03		Reference	
	500–1999	143	2.26		0.04 (-0.44–0.53)	0.862	2.10		0.06 (-0.42–0.55)	0.795
	2000–3499	118	2.55		0.34 (-0.25–0.92)	0.261	2.36		0.32 (-0.27–0.91)	0.283
	3500–4999	191	2.56		0.34 (-0.30–0.98)	0.299	2.34		0.31 (-0.34–0.95)	0.353
	≥5000	50	2.95		0.74 (-0.08–1.55) ^c,d^	0.076	2.79		0.75 (-0.07–1.57) ^c,d^	0.071

^a^ Minimally adjusted model: Adjusted for age, sex, job title, years of employment, daily working hours, low-back pain intensity the preceding 4 weeks prior to baseline, chronic low-back pain, and perceived stress during the two weeks prior to baseline. ^b^ Fully adjusted model: Minimally adjusted + BMI, leisure-time physical activity, smoking status, influence at work, access to work tools, role clarity, guidance, community and cohesion between colleagues, recognition, respectful relationship between leader and employees, and fairness. ^c^ Tendency (P=0.07-0.08) towards a statistically significant difference from 0-499 kg (reference). ^d^ Tendency (P=0.06-0.07) towards a statistically significant difference from 500–1999 kg.

## Discussion

The present study investigated whether LBP intensity, mental stress, and bodily fatigue were higher with progressively cumulated lifting loads in an exposure–response fashion after work and the following morning. Higher daily lifting loads were associated with higher LBP intensity after work and the following morning. Lifting ≥5000 kg was associated with elevated mental stress after work, while lifting 2000–3499 kg showed higher mental stress the following morning. No associations between lifting load and bodily fatigue was observed, although very high lifting loads (≥5000 kg) tended (P=0.071) to be associated with higher levels of bodily fatigue the following morning.

### Interpretation of findings

The present findings that higher lifting loads was associated with more intense LBP in warehouse workers elaborates on previous reports (4, 8, 9, 31). While cumulative lifting loads recorded within a day as well as across multiple working years are known to increase the risk of developing LBP (4, 8, 31), only few studies have investigated the day-to-day development in LBP (9, 32). Because supermarket and warehouse workers predominantly handle the same merchandise, the present observations as well as the day-to-day findings by Andersen and co-workers (9) of an exposure–response association between daily lifting loads and LBP intensity provide important knowledge about how to organize the work to prevent development of LBP. However, in the previous literature, we lack evidence about this association among warehouse workers who typically are exposed to higher lifting loads. Although the present investigation in this population did not reveal a linear exposure–response association, higher lifting loads were associated with higher LBP intensity after work and the following morning. In fact, LBP intensity was 1.26 points (NRS) higher after work when lifting ≥5000 kg, which is considerably above the minimal clinically important difference of ≥1 point on a NRS 0–10 (33). This observation underscores the importance of quantifying total daily lifting loads to prevent the development of LBP. Additionally, the elevated LBP intensity the following morning (0.97 points) closely approached the minimal clinically important difference of 1 NRS point.

LBP intensity registered at baseline (cf. table 1) as well as the absolute analysis estimates (cf. table 2, figure 2) clearly demonstrate warehouse workers to be markedly affected by LBP. In result, even small increases in LBP intensity could have detrimental effects on health in this population. A recent study among 69 000 workers from the general working population in Denmark reported that LBP intensity ≥3 points (NRS 0–10) increased the risk of long-term sickness absence within two years of register follow-up (34). Thus, the LBP levels observed in the present population seem to lie within an area where the risk of long-term sickness absence is elevated. Given that MSD are costly for both employees, employers, and society (6, 7), the present results underscore the importance of work environmental initiatives for reducing the lifting loads at work to prevent LBP and long-term sickness absence among warehouse workers. In support of this notion, we recently found heavy lifting tasks to increase the risk of long-term sickness absence in an exposure-response manner among 45 000 workers from the general working population in Denmark performing lifting work, ie, the heavier objects lifted, the higher risk of long-term sickness absence (35). Additionally, lifting for a large part of the workday increased the risk of long-term sickness absence (35). Consequently, initiatives to improve the physical working environment is crucial to prevent musculoskeletal health problems and maintain productivity.

The present study showed that very high lifting volumes (≥5000 kg) were associated with elevated mental stress after work, which elaborate on previous findings that physically demanding work can increase mental stress (19). The high lifting volumes may indicate a busy workday resulting in higher work pace/pressure, potentially affecting the mental stress level (15). Furthermore, because warehouse workers typically receive continuous instructions from the logistics system on what merchandise to pick, working at higher pace may result in higher lifting loads per workday. These factors comprise both physical and psychosocial job demands, leading to increased strain represented as mental stress (15). Besides experiencing increased strain, increased mental stress predicts musculoskeletal pain, including LBP (16, 17), which is consistent with the biopsychosocial origin of pain (36). Furthermore, the present study observed an association between lifting 2000–3499 kg and higher levels of mental stress the following morning. As this is the only lifting interval showing a statistically significant difference, the finding may be the result of statistical uncertainty or that workers in this lifting interval may have other mentally stressful job tasks. However, the observed stress estimates are relatively low, and therefore should be interpreted with caution.

Our hypothesis about an exposure–response association between cumulated lifting load and fatigue was not confirmed. However, an association tended to be observed for fatigue in the following morning when lifting ≥5000 kg (P=0.071). Previous studies have documented associations between physically demanding work, including lifting work, and increased bodily fatigue (10, 11, 37), whereas we lack evidence on associations between total daily lifting loads and fatigue in manual job conditions. Several factors may explain the lack of clear associations with fatigue in the present study. The 12-hours interval between the daily questionnaires may result in some workers ending their workday 3–4 hours before receiving the ‘after work’ questionnaire. This could affect the results because participants may have restituted from fatigue during the initial post work hours. Furthermore, the feeling of pain could potentially suppress the perception of fatigue after work, resulting in a lower rating of perceived fatigue. Further, the perception of fatigue may remain partially suppressed in the following morning, which may explain the borderline significance. Together with the low sample size, these factors may contribute to the lack of clear associations between lifting load and fatigue.

The daily mean lifting loads observed in the present study were higher than previously reported for supermarket workers (9), and the standard deviation shows a large variation in the lifting loads (cf. table 1). This variability was mainly due to a high number of observations with low lifting loads (0–499 kg). Furthermore, workers with high physical capacity, who may tolerate high lifting loads, could represent a relatively large part of the total observations in the ≥5000 kg interval, suggestive of a healthy worker effect (38). Conversely, the large inter-individual variations in lifting loads presently observed could represent a potential to homogenize the lifting tasks and volume more equally between warehouse workers.

A previous study among scaffold workers found the use of company records to be an accurate and cost-efficient method assessing the physical exposure objectively, finding an explained variance of 77–92% in the number of lifting tasks (26). Furthermore, a study from our lab used company records to investigate associations between total daily lifting loads and day-to-day changes in LBP intensity (9). Previously, prospective associations between physical job demands and health outcomes have used questionnaires to estimate the physical exposure (3, 23, 24), which comprises well-known methodological limitations. In contrast, highly detailed technical measurements of the physical workload during workdays by means of accelerometers, 3D motion capture, video-observations, and force measurements (8, 39–42), are time-consuming, expensive, and require a high level of technical expertise. Besides being more cost-efficient and providing objective data on cumulated exposure, warehouses (and other workplaces) hold company records, which support the potential to plan the work and distribute the lifting loads more equally between the workers to reduce the prevalence of very high lifting loads.

### Limitations and strengths

The present study comprises both limitations and strengths. The relatively low sample size (N=85) represents a limitation of the study. Approaching the planned sample size (2) would increase the statistical power and allow a narrowing of the load categorizations. Conversely, the repeated measure design increases the statistical power, and the current study sample allowed analyzing the exposure variable categorically. On the other hand, employing a repeated measures design as in the present study carries the risk of a relatively high within-worker variance (43). In such instances, a fixed-effect model would be more viable than a random-effects model, given its ability to negate time-independent unmeasured confounding, effectively allowing the worker to serve as his/her own control (43). We therefore calculated the within- and between-worker variance of the exposure measure (load lifted per day), which showed that the total variance was distributed in 22% within-worker variance and 78% between-worker variance. This affirmed that a mixed model would be more appropriate than a fixed model, leveraging the repeated measures study design to its fullest potential. Questionnaire data are prone to common-method variance where a person’s mood, health status, and interpretation can influence the replies (25). By contrast, using company records to measure the exposure objectively represents a methodological strength of the study. Because the workers subsequently replied questions about perceived work-related symptoms (outcome variables) after work and the following morning, the study design was able to separate the exposure and outcome in regards to source and time. Furthermore, the repeated measures design with twice-daily questionnaires for 21 days eliminated recall bias. As a limitation with the present study, however, only total lifting load was quantified. Warehouse workers often perform other work tasks, which could have contributed to decrease the estimates and widen the CI. Furthermore, during stocking of pallets warehouse workers are exposed to worsening factors in terms of excessive lifting heights, asymmetrical lifting, and awkward work postures, which increases the loads on muscles and joints (41, 42). Using validated NRS for LBP (28) and fatigue (29, 30) strengthens the study. The scale used to measure daily mental stress is not validated, but 0–10 scales are frequently used to detect changes, eg, daily changes in mental stress using Borg CR 10 (44). Lastly, we adjusted for relatively many relevant confounders, which may lead to overadjustment, although we carefully kept the confounders to a minimum.

### Concluding remarks

This study found occupational lifting to affect both physical and psychological work-related symptoms. Using company records as an objective measure of physical exposure, higher total daily lifting loads were associated with elevated LBP intensity after work and the following morning, with high lifting loads showing clinically significant elevations in LBP. Furthermore, higher lifting loads were associated with higher mental stress after work, while no statistically significant associations were observed between lifting loads and bodily fatigue. Warehouse administrations could use company records of loads lifted to organize the distribution of warehouse work among employees in a manner that reduce the high lifting loads and equalize total lifting work between workers to prevent musculoskeletal health problems and promote physical and psychological well-being.

## Supplementary material

Supplementary material

## Data Availability

Data is available upon reasonable request. The authors encourage collaboration and use of the data by other researchers. Data is stored on a secure server of the National Research Centre for the Working Environment, and researchers interested in using the data for scientific purposes should contact Rúni Bláfoss (rub@nfa.dk).

## Protection of research participants

All data are stored on a secure server at the National Research Centre for the Working Environment and only available in anonymized format to the researchers.

## References

[1] NRCWE. Work Environment & Health in Denmark [Internet]. National Research Centre for the Working Environment (NRCWE); 2018 [cited 2020 Oct 29]. Available from: https://at.dk/arbejdsmiljoe-i-tal/analyser-og-publikationer/arbejdsmiljoe-og-helbred-2012-2018/

[2] Bláfoss R, Aagaard P, Andersen LL. Physical and psychosocial work environmental risk factors of low-back pain: protocol for a 1 year prospective cohort study. BMC Musculoskelet Disord 2019 Dec;20(1):626. 10.1186/s12891-019-2996-z 31881868 PMC6933884

[3] da Costa BR, Vieira ER. Risk factors for work-related musculoskeletal disorders: A systematic review of recent longitudinal studies. Am J Ind Med 2010 Mar;53(3):285–323. 10.1002/ajim.20750 19753591

[4] Coenen P, Gouttebarge V, van der Burght AS, van Dieën JH, Frings-Dresen MH, van der Beek AJ et al. The effect of lifting during work on low back pain: a health impact assessment based on a meta-analysis. Occup Environ Med 2014 Dec;71(12):871–7. 10.1136/oemed-2014-102346 25165395

[5] Govaerts R, Tassignon B, Ghillebert J, Serrien B, De Bock S, Ampe T et al. Prevalence and incidence of work-related musculoskeletal disorders in secondary industries of 21st century Europe: a systematic review and meta-analysis. BMC Musculoskelet Disord 2021 Aug;22(1):751. 10.1186/s12891-021-04615-9 34465326 PMC8408961

[6] Bevan S. Economic impact of musculoskeletal disorders (MSDs) on work in Europe. Best Pract Res Clin Rheumatol 2015 Jun;29(3):356–73. 10.1016/j.berh.2015.08.002 26612235

[7] Sundhedsstyrelsen. Sygdomsbyrden i Danmark - sygdomme [Internet]. Copenhagen: Danish Health Authority; 2022 [cited 2023 Mar 3] p. 1–460. Available from: https://www.sst.dk/-/media/Udgivelser/2023/Sygdomsbyrden-2023/Sygdomme-Sygdomsbyrden-2023.ashx

[8] Coenen P, Kingma I, Boot CR, Bongers PM, van Dieën JH. Cumulative mechanical low-back load at work is a determinant of low-back pain. Occup Environ Med 2014 May;71(5):332–7. 10.1136/oemed-2013-101862 24676271

[9] Andersen LL, Fallentin N, Ajslev JZ, Jakobsen MD, Sundstrup E. Association between occupational lifting and day-to-day change in low-back pain intensity based on company records and text messages. Scand J Work Environ Health 2017 Jan;43(1):68–74. 10.5271/sjweh.3592 27611578

[10] Mital A, Foononi-Fard H, Brown ML. Physical fatigue in high and very high frequency manual materials handling: perceived exertion and physiological indicators. Hum Factors 1994 Jun;36(2):219–31. 10.1177/001872089403600204 8070788

[11] Januario LB, Stevens ML, Mathiassen SE, Holtermann A, Karstad K, Hallman DM. Combined Effects of Physical Behavior Compositions and Psychosocial Resources on Perceived Exertion Among Eldercare Workers. Ann Work Expo Health 2020 Nov;64(9):923–35. 10.1093/annweh/wxaa079 32729914 PMC7751016

[12] Hanvold TN, Wærsted M, Mengshoel AM, Bjertness E, Stigum H, Twisk J et al. The effect of work-related sustained trapezius muscle activity on the development of neck and shoulder pain among young adults. Scand J Work Environ Health 2013 Jul;39(4):390–400. 10.5271/sjweh.3357 23494255

[13] Andersen LL, Clausen T, Burr H, Holtermann A. Threshold of musculoskeletal pain intensity for increased risk of long-term sickness absence among female healthcare workers in eldercare. PLoS One 2012;7(7):e41287. 10.1371/journal.pone.0041287 22911772 PMC3401109

[14] Sagherian K, Geiger-Brown J, Rogers VE, Ludeman E. Fatigue and risk of sickness absence in the working population: A systematic review and meta-analysis of longitudinal studies. Scand J Work Environ Health 2019 Jul;45(4):333–45. 10.5271/sjweh.3819 30937459

[15] Bakker AB, Demerouti E. The Job Demands‐Resources model: state of the art. J Manag Psychol 2007 Apr;22(3):309–28. 10.1108/02683940710733115

[16] Amiri S, Behnezhad S. Is job strain a risk factor for musculoskeletal pain? A systematic review and meta-analysis of 21 longitudinal studies. Public Health 2020 Apr;181:158–67. 10.1016/j.puhe.2019.11.023 32059156

[17] Vinstrup J, Jakobsen MD, Andersen LL. Perceived Stress and Low-Back Pain Among Healthcare Workers: A Multi-Center Prospective Cohort Study. Front Public Health 2020 Aug;8:297. 10.3389/fpubh.2020.00297 32850571 PMC7431956

[18] McEwen BS. Protective and damaging effects of stress mediators: central role of the brain. Dialogues Clin Neurosci 2006;8(4):367–81. 10.31887/DCNS.2006.8.4/bmcewen 17290796 PMC3181832

[19] Dėdelė A, Miškinytė A, Andrušaitytė S, Bartkutė Ž. Perceived Stress among Different Occupational Groups and the Interaction with Sedentary Behaviour. Int J Environ Res Public Health 2019 Nov;16(23):4595. 10.3390/ijerph16234595 31756951 PMC6926860

[20] Courvoisier DS, Genevay S, Cedraschi C, Bessire N, Griesser-Delacretaz AC, Monnin D et al. Job strain, work characteristics and back pain: a study in a university hospital. Eur J Pain 2011 Jul;15(6):634–40. 10.1016/j.ejpain.2010.11.012 21186129

[21] Andersen LL, Pedersen J, Sundstrup E, Thorsen SV, Rugulies R. High physical work demands have worse consequences for older workers: prospective study of long-term sickness absence among 69 117 employees. Occup Environ Med 2021 Nov;78(11):829–34. 10.1136/oemed-2020-107281 33972376 PMC8526881

[22] Andersen LL, Vinstrup J, Thorsen SV, Pedersen J, Sundstrup E, Rugulies R. Combined psychosocial work factors and risk of long-term sickness absence in the general working population: prospective cohort with register follow-up among 69 371 workers. Scand J Work Environ Health 2022 Sep;48(7):549–59. 10.5271/sjweh.4035 35647686 PMC10539106

[23] Sterud T. Work-related mechanical risk factors for long-term sick leave: a prospective study of the general working population in Norway. Eur J Public Health 2014 Feb;24(1):111–6. 10.1093/eurpub/ckt072 23748849

[24] Andersen LL, Vinstrup J, Sundstrup E, Skovlund SV, Villadsen E, Thorsen SV. Combined ergonomic exposures and development of musculoskeletal pain in the general working population: A prospective cohort study. Scand J Work Environ Health 2021 May;47(4):287–95. 10.5271/sjweh.3954 33749799 PMC8091072

[25] Podsakoff PM, MacKenzie SB, Lee JY, Podsakoff NP. Common method biases in behavioral research: a critical review of the literature and recommended remedies. J Appl Psychol 2003 Oct;88(5):879–903. 10.1037/0021-9010.88.5.879 14516251

[26] van der Beek AJ, Mathiassen SE, Burdorf A. Efficient assessment of exposure to manual lifting using company data. Appl Ergon 2013 May;44(3):360–5. 10.1016/j.apergo.2012.09.006 23069188

[27] von Elm E, Altman DG, Egger M, Pocock SJ, Gøtzsche PC, Vandenbroucke JP; STROBE Initiative. The Strengthening the Reporting of Observational Studies in Epidemiology (STROBE) statement: guidelines for reporting observational studies. J Clin Epidemiol 2008 Apr;61(4):344–9. 10.1016/j.jclinepi.2007.11.008 18313558

[28] Downie WW, Leatham PA, Rhind VM, Wright V, Branco JA, Anderson JA. Studies with pain rating scales. Ann Rheum Dis 1978 Aug;37(4):378–81. 10.1136/ard.37.4.378 686873 PMC1000250

[29] Gladman D, Nash P, Goto H, Birt JA, Lin CY, Orbai AM et al. Fatigue numeric rating scale validity, discrimination and responder definition in patients with psoriatic arthritis. RMD Open 2020 Jan;6(1):e000928. 10.1136/rmdopen-2019-000928 31958274 PMC7046948

[30] Machado MO, Kang NC, Tai F, Sambhi RD, Berk M, Carvalho AF et al. Measuring fatigue: a meta-review. Int J Dermatol 2021 Sep;60(9):1053–69. 10.1111/ijd.15341 33301180

[31] Brauer C, Mikkelsen S, Pedersen EB, Møller KL, Simonsen EB, Koblauch H et al. Occupational lifting predicts hospital admission due to low back pain in a cohort of airport baggage handlers. Int Arch Occup Environ Health 2020 Jan;93(1):111–22. 10.1007/s00420-019-01470-z 31451926 PMC6989598

[32] Burström L, Jonsson H, Björ B, Hjalmarsson U, Nilsson T, Reuterwall C et al. Daily text messages used as a method for assessing low back pain among workers. J Clin Epidemiol 2016 Feb;70:45–51. 10.1016/j.jclinepi.2015.08.011 26342444

[33] Dworkin RH, Turk DC, Wyrwich KW, Beaton D, Cleeland CS, Farrar JT et al. Interpreting the clinical importance of treatment outcomes in chronic pain clinical trials: IMMPACT recommendations. J Pain 2008 Feb;9(2):105–21. 10.1016/j.jpain.2007.09.005 18055266

[34] Skovlund SV, Bláfoss R, Calatayud J, López Bueno R, Sundstrup E, Andersen LL. Musculoskeletal pain intensity and risk of long-term sickness absence in the general working population: A prospective cohort study with register follow-up. Prev Med 2023 Sep;174:107636. 10.1016/j.ypmed.2023.107636 37473925

[35] Bláfoss R, Skovlund SV, Skals S, Sundstrup E, López-Bueno R, Calatayud J et al. Duration and intensity of occupational lifting and risk of long-term sickness absence: prospective cohort study with register follow-up among 45 000 workers. Scand J Work Environ Health 2023 May;49(4):283–92. 10.5271/sjweh.4085 36881789 PMC10713984

[36] Engel GL. The need for a new medical model: a challenge for biomedicine. Science 1977 Apr;196(4286):129–36. 10.1126/science.847460 847460

[37] Brambilla C, Lavit Nicora M, Storm F, Reni G, Malosio M, Scano A. Biomechanical Assessments of the Upper Limb for Determining Fatigue, Strain and Effort from the Laboratory to the Industrial Working Place: A Systematic Review. Bioengineering (Basel) 2023 Apr;10(4):445. 10.3390/bioengineering10040445 37106632 PMC10135542

[38] McMichael AJ. Standardized mortality ratios and the “healthy worker effect”: scratching beneath the surface. J Occup Med 1976 Mar;18(3):165–8. 10.1097/00043764-197603000-00009 1255276

[39] Jakobsen MD, Sundstrup E, Brandt M, Persson R, Andersen LL. Estimation of physical workload of the low-back based on exposure variation analysis during a full working day among male blue-collar workers. Cross-sectional workplace study. Appl Ergon 2018 Jul;70:127–33. 10.1016/j.apergo.2018.02.019 29866301

[40] Brandt M, Madeleine P, Samani A, Ajslev JZ, Jakobsen MD, Sundstrup E et al. Effects of a Participatory Ergonomics Intervention With Wearable Technical Measurements of Physical Workload in the Construction Industry: Cluster Randomized Controlled Trial. J Med Internet Res 2018 Dec;20(12):e10272. 10.2196/10272 30567694 PMC6315250

[41] Skals S, Bláfoss R, Andersen MS, de Zee M, Andersen LL. Manual material handling in the supermarket sector. Part 1: joint angles and muscle activity of trapezius descendens and erector spinae longissimus. Appl Ergon 2021 Apr;92:103340. 10.1016/j.apergo.2020.103340 33340719

[42] Skovlund SV, Bláfoss R, Skals S, Jakobsen MD, Andersen LL. Technical field measurements of muscular workload during stocking activities in supermarkets: cross-sectional study. Sci Rep 2022 Jan;12(1):934. 10.1038/s41598-022-04879-8 35042941 PMC8766430

[43] van de Ven D, Robroek SJ, Oude Hengel KM, van Zon SK, Brouwer S, Ots P et al. Associations of within-individual changes in working conditions, health behaviour and BMI with work ability and self-rated health: a fixed effects analysis among Dutch workers. BMJ Open 2022 Apr;12(4):e058574. 10.1136/bmjopen-2021-058574 35487715 PMC9058761

[44] Hallman DM, Ekman AH, Lyskov E. Changes in physical activity and heart rate variability in chronic neck-shoulder pain: monitoring during work and leisure time. Int Arch Occup Environ Health 2014;87(7):735–44. 10.1007/s00420-013-0917-2 24162088

