# Supplementary material for: Association of objectively measured lifting load with low-back pain, stress, and fatigue: A prospective cohort study
Source: Scand J Work Environ Health. 2023 Dec 18;50(1):11–21. doi: 10.5271/sjweh.4127 (PMC10917448; doi:10.5271/sjweh.4127)
Supplement: Supplementary material [file SJWEH-50-11-S001.pdf]

Association of objectively measured lifting load with low-back pain, stress, and fatigue: A prospective cohort study[1]

by Rúni Bláfoss, MSc,[2] Per Aagaard, PhD, Thomas Clausen, PhD, Lars L Andersen, PhD

1. *Supplementary Material*
2. *Correspondence to: Rúni Bláfoss, National Research Centre for the Working Environment, Lersø Parkalle 105, DK-2100 Copenhagen, Denmark. [E-mail: rub@nfa.dk]*

Methods

Amendments to the study protocol

Compared to our study protocol (2), several amendments have been conducted due to recruitment challenges and the Covid-19 pandemic from 2020-2022. First, implementation of new logistic software and building of new warehouse terminals challenged the recruitment of warehouses and warehouse workers. Second, during the pandemic, the retail chains were unable to cooperate due to risk of infection and higher demands for supply. Third, after the pandemic, some warehouses did not have the resources to participate in research, because they lacked employees due to inflation.

Because of these factors, the project period was extended ~1 year, i.e. the project period does not follow the time schedule specified in our study protocol (2). To attain a sufficient number of participants, during the recruitment phase, we recruited additional types of warehouses within the retail industry. At last, not all warehouses could provide company records about daily lifting loads for each warehouse worker. All these amendments resulted in a smaller sample size and a revised publication plan than initially outlined in the protocol article (2).

Supplementary Figure 1. Association between total lifting load during the workday and low-back pain intensity (delta estimates) after work (A) and the following morning (B) in the fully adjusted model (NRS 0-10).

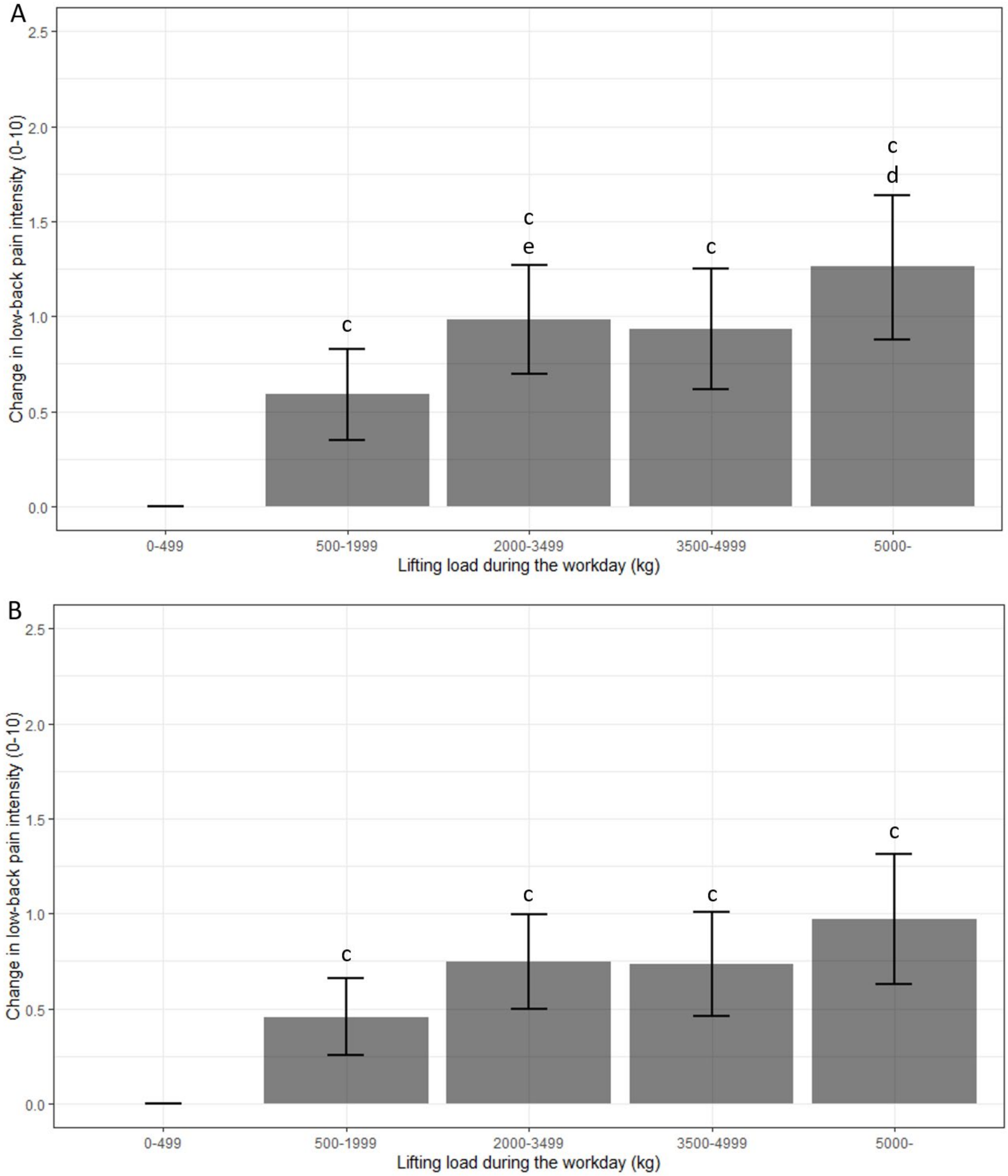

[c] Statistically significant different from 0-499 kg (reference). [d] Statistically significant different from 500-1999 kg. [e] Tendency towards a statistically significant difference from 500-1999 kg (p=0.062).

Supplementary Figure 2. Association between total lifting load during the workday and mental stress (absolute estimates) after work (A) and the following morning (B) in the fully adjusted model.

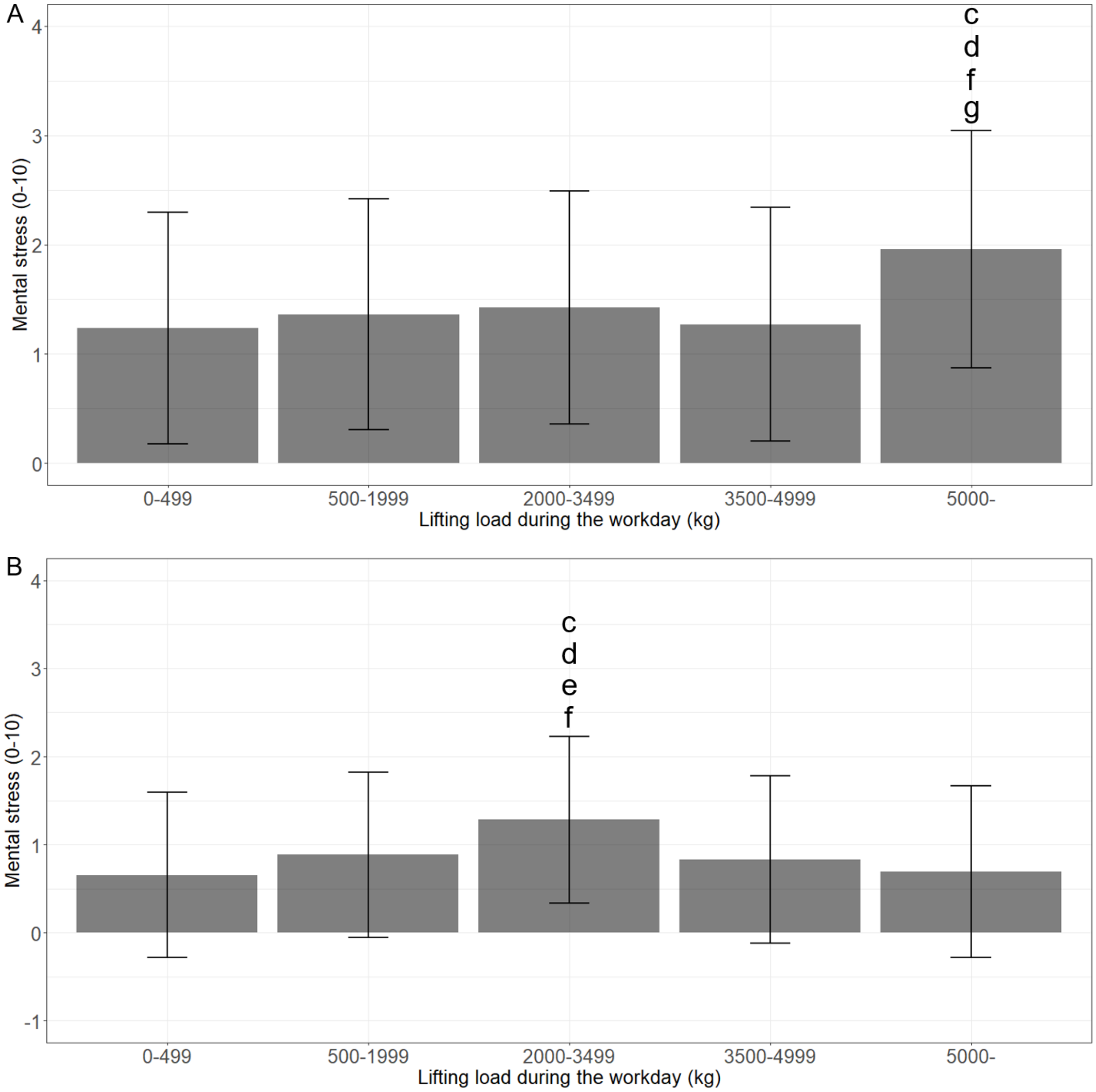

[c] Statistically significant different from 0-499 kg (reference).

[d] Statistically significant different from 500–1999 kg.

[e] Statistically significant different from 2000–3499 kg.

[f] Statistically significant different from 3500–4999 kg.

[g] Statistically significant different from ≥5000 kg.

Supplementary Figure 3. Association between total lifting load during the workday and mental stress (delta estimates) after work (A) and the following morning (B) in the fully adjusted model.

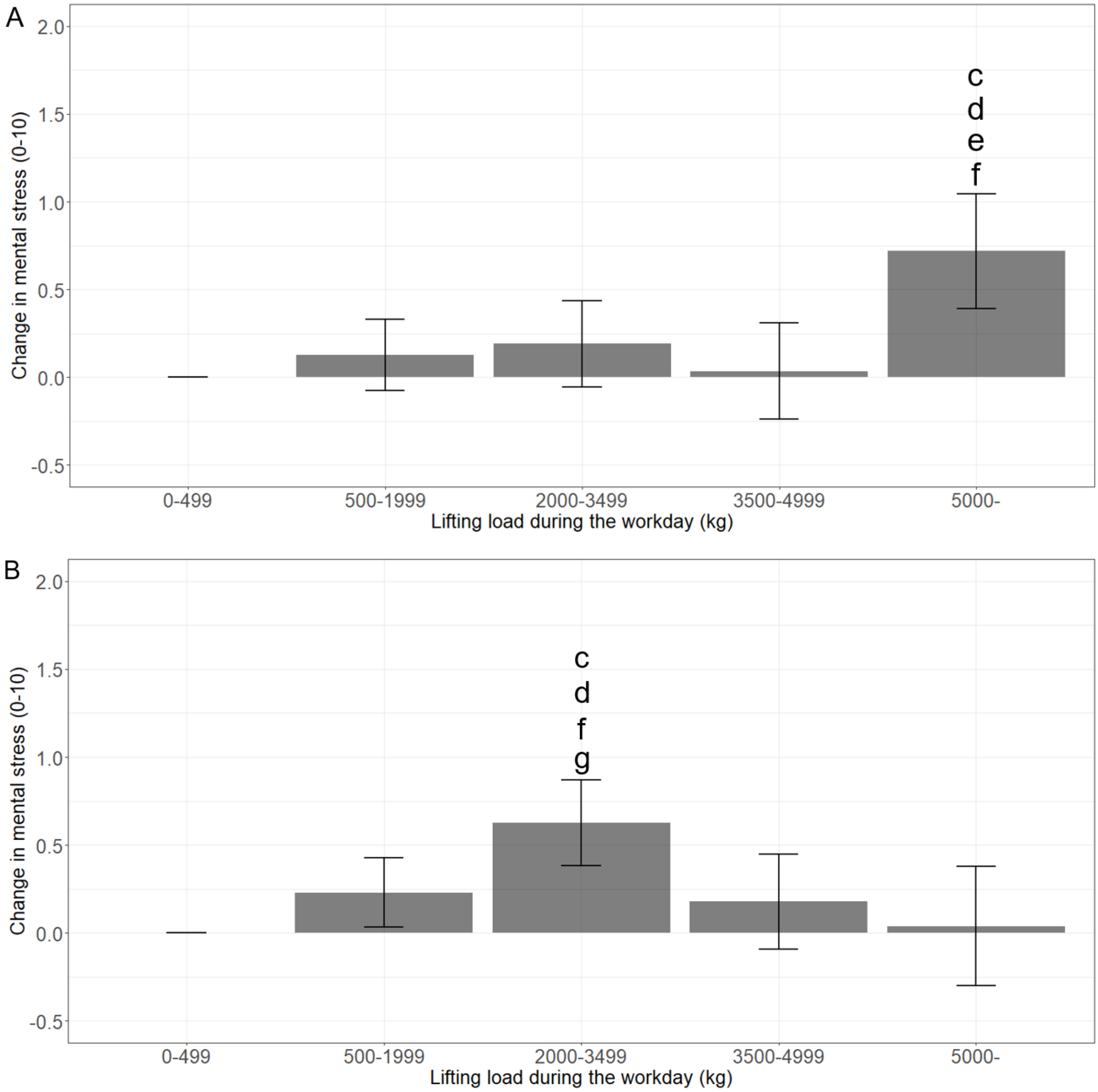

[c] Statistically significant different from 0-499 kg (reference).
[d] Statistically significant different from 500–1999 kg.
[e] Statistically significant different from 2000–3499 kg.
[f] Statistically significant different from 3500–4999 kg.
[g] Statistically significant different from ≥5000 kg.

Supplementary Figure 4. Association between total lifting load during the workday and bodily fatigue (absolute estimates) after work (A) and the following morning (B) in the fully adjusted model (NRS – Fatigue 0-10).

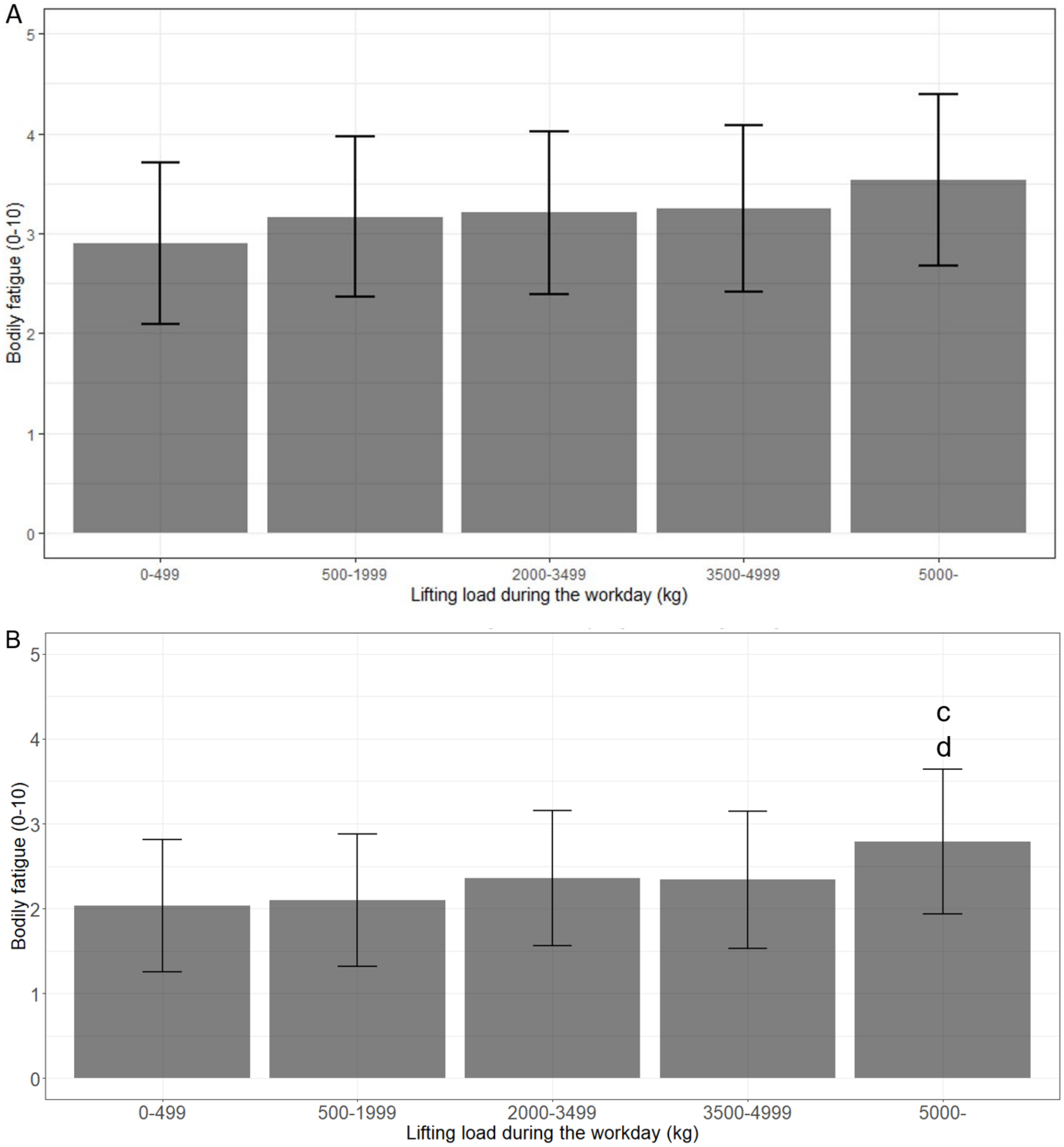

[c] Tendency (P=0.07-0.08) towards a statistically significant difference from 0-499 kg (reference).
[d] Tendency (P=0.06-0.07) towards a statistically significant difference from 500–1999 kg.

Supplementary Figure 5. Association between total lifting load during the workday and bodily fatigue (delta estimates) after work (A) and the following morning (B) in the fully adjusted model (NRS – Fatigue 0-10).

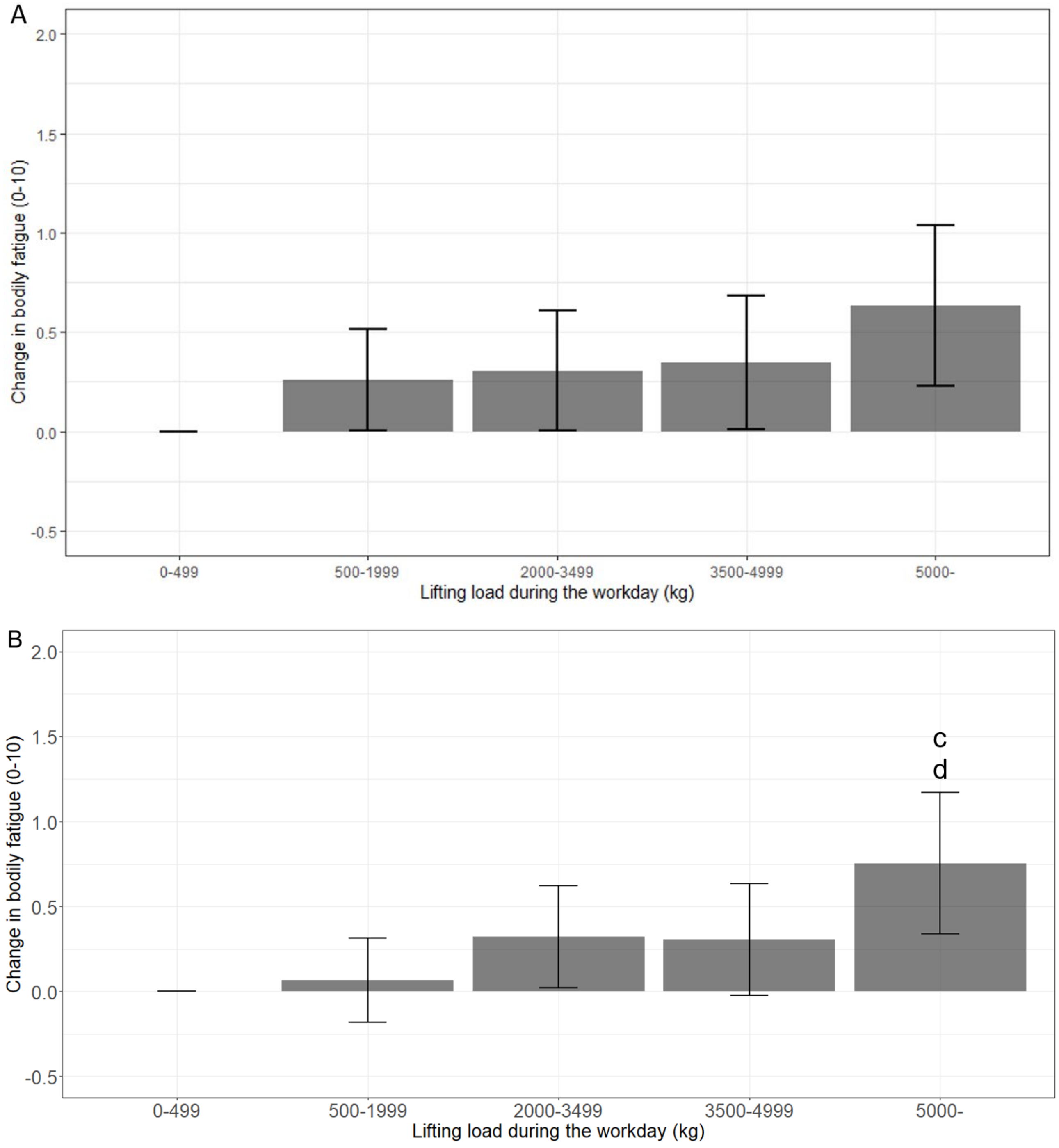

[c] Tendency (P=0.07-0.08) towards a statistically significant difference from 0-499 kg (reference).
[d] Tendency (P=0.06-0.07) towards a statistically significant difference from 500–1999 kg.
